# Morphological Alterations in Newly Born Dentate Gyrus Granule Cells That Emerge after *Status Epilepticus* Contribute to Make Them Less Excitable

**DOI:** 10.1371/journal.pone.0040726

**Published:** 2012-07-11

**Authors:** Julián Tejada, Gabriel M. Arisi, Norberto García-Cairasco, Antonio C. Roque

**Affiliations:** 1 Departamento de Física, Faculdade de Filosofia, Ciências e Letras de Ribeirão Preto, Universidade de São Paulo, Ribeirão Preto, São Paulo, Brazil; 2 Departamento de Fisiologia, Faculdade de Medicina de Ribeirão Preto, Ribeirão Preto, São Paulo, Brazil; Georgia State University, United States of America

## Abstract

Computer simulations of external current stimulations of dentate gyrus granule cells of rats with *Status Epilepticus* induced by pilocarpine and control rats were used to evaluate whether morphological differences alone between these cells have an impact on their electrophysiological behavior. The cell models were constructed using morphological information from tridimensional reconstructions with Neurolucida software. To evaluate the effect of morphology differences alone, ion channel conductances, densities and distributions over the dendritic trees of dentate gyrus granule cells were the same for all models. External simulated currents were injected in randomly chosen dendrites belonging to one of three different areas of dentate gyrus granule cell molecular layer: inner molecular layer, medial molecular layer and outer molecular layer. Somatic membrane potentials were recorded to determine firing frequencies and inter-spike intervals. The results show that morphologically altered granule cells from pilocarpine-induced epileptic rats are less excitable than control cells, especially when they are stimulated in the inner molecular layer, which is the target area for mossy fibers that sprout after pilocarpine-induced cell degeneration. This suggests that morphological alterations may act as a protective mechanism to allow dentate gyrus granule cells to cope with the increase of stimulation caused by mossy fiber sprouting.

## Introduction

The effects of *Status Epilepticus* (SE) on neurogenesis of the dentate gyrus (DG) granule cells (GCs) have been widely described [1], [2]. Generally those studies report morphological alterations that include modifications of the branching patterns of apical dendrites, presence of basal dendrites that are projected towards the DG hilus, ectopic localization and even dysmorphic presentations [1], [3]–[8].

These cells also present differences in the number of dendritic spines on the basal dendrites [7], [9], [10]. In addition, the axon of mature and new DG cells can innervate the inner molecular layer of DG in a phenomenon called mossy fiber sprouting, creating recurrent excitatory connections [1], [6], [10]–[12].

Regarding the electrophysiological behavior of GCs in SE rats, there are conflicting results. Some studies [3], [13] report no differences in electrical activity between GCs from control and electrical kindling-, pilocarpine (PILO)- or kainate-induced epileptic animals. On the other hand, there is evidence of either hyperexcitability [14] or hipoexcitability [15] of GCs in animal models of epilepsy.

The way in which changes in the morphology of DG GCs are integrated into the DG and how they can eventually affect the spontaneous emergence of seizures is still unknown. A possible effect of the morphological changes would be that they, combined with reduced inhibition, contribute to turn the cells more excitable [14], [16]. However, it would also be possible that they have the opposite effect, i.e. they tend to reduce excitability as a mechanism to counterbalance the dysfunctional effects of SE [15].

Therefore, new approaches are necessary to elucidate the consequences of morphological alterations and their associated mechanisms. In this context, computational modeling presents a quite good alternative to generate hypotheses and guide future research. Different biologically detailed models of DG GCs have been constructed, from single cell models in normal conditions [17]–[19] to networks to study the effects of axonal fiber sprouting in epilepsy [16], [20], [21] or the contribution of adult neurogenesis to memory formation [22], [23].

These computational studies [16], [20], [21] have shown that morphological changes, such as mossy fiber sprouting, combined with ion channel and connectivity alterations provoke dentate hyperexcitability. However, the effects of changes in the dendritic cell morphology have not yet been studied. It is known that changes in dendritic morphology alter the cellular spiking behavior [24]–[26] and an open question is what would be the consequences of morphological dendritic changes on GC excitability.

For this reason, in the present study we constructed computational models of morphologically reconstructed newborn DG GCs from rats that had SE induced by PILO and from control rats to compare their firing responses when stimulated by simulated current injections in their dendritic trees. The source of our morphological data was a set of Neurolucida-based tridimensional reconstructions of rat newly born doublecortin-positive DG GCs [6].

To construct our cell models, we assumed that the ionic channels, their maximal conductance densities and their distributions over the different areas of the DG GC dendritic tree are the same for all cells. This would allow us to predict possible effects that changes in dendritic arborization alone can have on the electrical behavior of the cells.

Our results show that newborn DG GCs from rats with SE induced by PILO are less excitable than newborn DG GCs from control rats. Based on this finding we predict that morphological alterations in newborn DG GCs caused by SE induced by PILO can provoke changes in the firing behavior of these cells. Moreover, these changes tend to cause opposite effects on the activity of newborn DG GCs than the ones caused by PILO.

## Results

**Figure 1:**
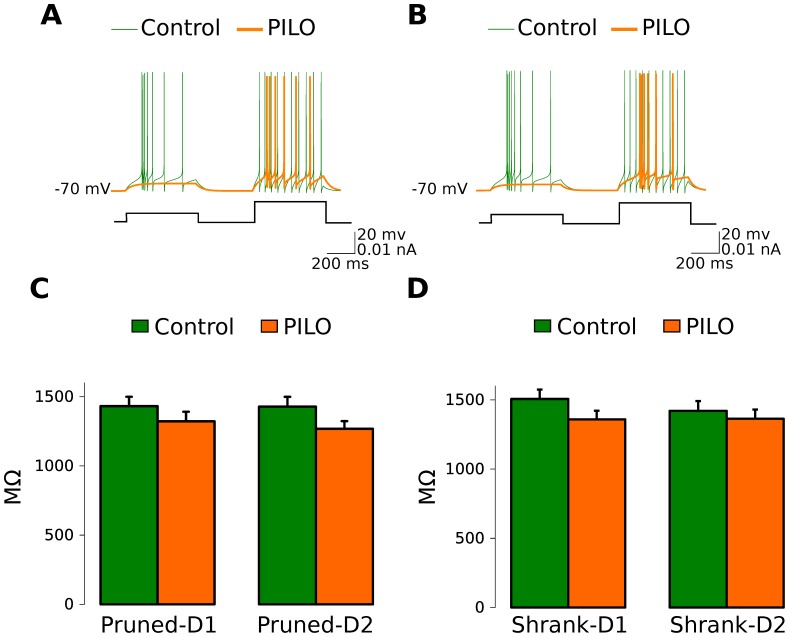
Representative firing traces of the control and PILO model cells and input resistance values measured in the simulations. **A**. Firing traces for pruned-distance 1 cell models stimulated with three electrodes in the MML. The green curve is for the Control-GC n09-cont02-sl2 and the orange curve is for the PILO-GC n33-r05-01-s14 model. **B**. Firing traces for the same cell models as in A with the same stimulation protocol but built using the pruned-distance 2 criterion. **C**. Average input resistance for pruned-distance 1 and distance 2 cell models. **D**. Average input resistance for shrank-distance 1 and distance 2 cell models. Error bars represent standard error.

In general the compartmental cell models reproduced the responses of actual DG GCs to current step injection with a sequence of spikes with adaptation and no burst [14] (Figures 1A–B). Values of input resistance are in the gigaohm range as expected for young GCs [27] and are similar for both Control and PILO GCs in each one of the different models: pruned-distance-1, pruned-distance-2, shrank-distance-1, and shrank-distance-2 (Figures 1C–D).

**Figure 2:**
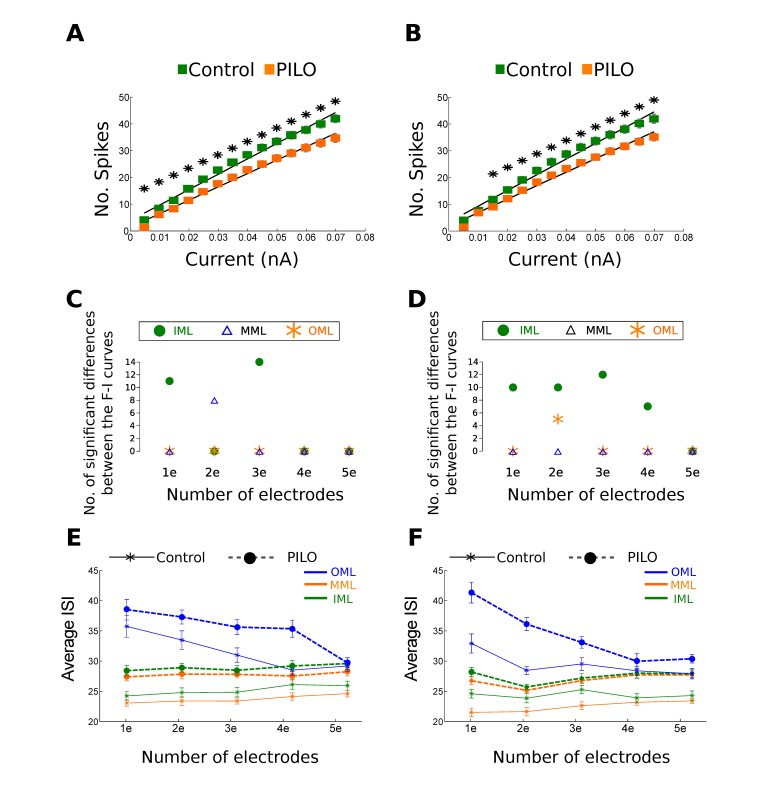
Summary with the main results displayed by the pruned models. **A**. F–I curves for pruned-distance 1 cell models stimulated with three electrodes in the MML. The error bars represent standard error. **B**. F–I curves for pruned-distance 2 cell models stimulated with three electrodes in the MML. Asterisks represent significant differences between Control-GC and PILO-GC models. Error bars represent standard error. **C**. Number of significant differences between the F–I curves of pruned-distance 1 Control-GC and PILO-GC models for each region of stimulation (IML, MML and OML) and for each number of electrodes (1e = one electrode, 2e = two electrodes, and so on). **D**. Number of significant differences between the F–I curves of pruned-distance 2 Control-GC and PILO-GC models for each region of stimulation (IML, MML and OML) and for each number of electrodes (1e = one electrode, 2e = two electrodes, and so on). **E**. Average inter-spike interval for pruned-distance 1 cell models estimated for each region of stimulation (IML, MML and OML) and for each number of electrodes (1e = one electrode, 2e = two electrodes, and so on). **F**. Average inter-spike interval for pruned-distance 2 cell models estimated for each region of stimulation (IML, MML and OML) and for each number of electrodes (1e = one electrode, 2e = two electrodes, and so on).

**Figure 3:**
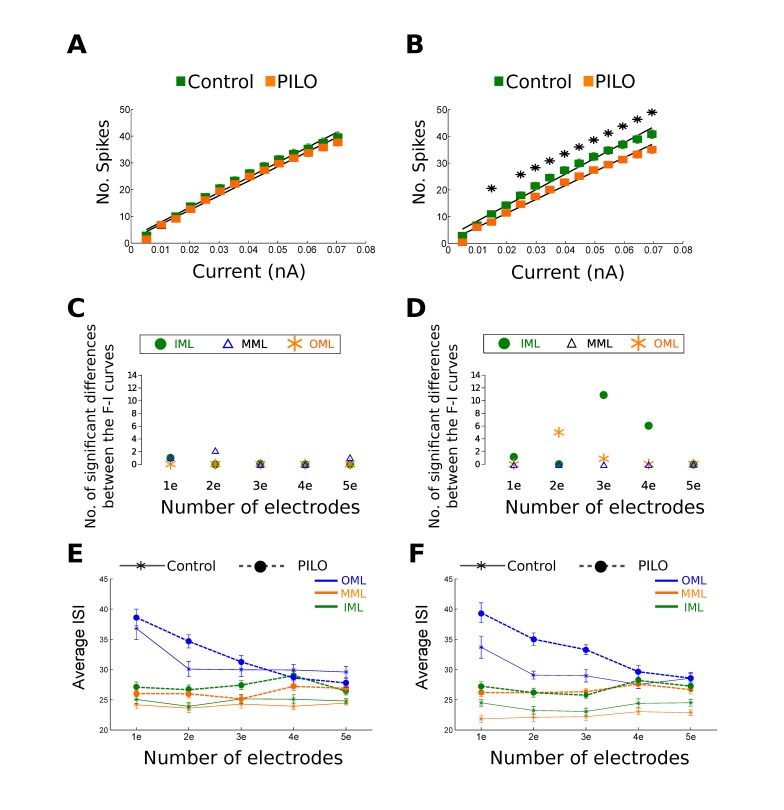
Summary with the main results displayed by the shrank models. **A**. F–I curves for shrank-distance 1 cell models stimulated with three electrodes in the MML. The error bars represent standard error. **B**. F–I curves for shrank-distance 2 cell models stimulated with three electrodes in the MML. Asterisks represent significant differences between Control-GC and PILO-GC models. Error bars represent standard error. **C**. Number of significant differences between the F–I curves of shrank-distance 1 Control-GC and PILO-GC models for each region of stimulation (IML, MML and OML) and for each number of electrodes (1e = one electrode, 2e = two electrodes, and so on). **D**. Number of significant differences between the F–I curves of shrank-distance 2 Control-GC and PILO-GC models for each region of stimulation (IML, MML and OML) and for each number of electrodes (1e = one electrode, 2e = two electrodes, and so on). **E**. Average inter-spike interval for shrank-distance 1 cell models estimated for each region of stimulation (IML, MML and OML) and for each number of electrodes (1e = one electrode, 2e = two electrodes, and so on). **F**. Average inter-spike interval for shrank-distance 2 cell models estimated for each region of stimulation (IML, MML and OML) and for each number of electrodes (1e = one electrode, 2e = two electrodes, and so on).

The cell models in the PILO groups show smaller firing frequencies and higher inter-spike intervals than control cells. This pattern was observed in all groups of cell models (pruned-shrank, distance 1-distance 2 cells), with minor differences depending on the number of electrodes or the layer in which the cell was stimulated (see Figures 2 and 3).

Most of the significant differences in the number of spikes in the F–I curves occur for the pruned models, and they are concentrated mainly in the stimulations made in the IML regardless of the number of electrodes (see Figure 2). In particular, the pruned-distance 2 models (see Figures 2B, D and F) exhibit significant spike number differences for the IML stimulations with almost any number of electrodes (the only non-significant case being with five electrodes). On the other hand, the F–I curves of the stimulations in the MML and OML only show differences between PILO and control cells for some electrode configuration, one in the MML layer for pruned-distance 1 models and two electrodes, one in the OML layer for pruned-distance 2 models and two electrodes, one in the MML layer for shrank-distance 1 models and five electrodes, and one in the OML layer for shrank-distance 2 models and two electrodes (see Figures 2C–D and 3C–D).

The inter-spike intervals show a similar pattern to the F–I curves, with differences between PILO and control cells occurring mostly for current injections in the IML regardless of the number of electrodes (see Figures 2E–F and 3E–F). Similarly to the F–I curves the inter-spike interval differences are concentrated mostly in the pruned-distance 2 cell models. However, the inter-spike intervals show differences between PILO and control cells in the MML and OML layer for more electrode configuration than the F–I curves (see Figures 2E–F and 3E–F).

## Discussion

This study investigated the effect that morphology differences alone can have on the spiking behavior of biophysically realistic multicompartmental models [28] of morphologically reconstructed granule cells from epileptic and control rat DG [6]. The models had the same types of ion channels and the same distributions of conductance densities over their dendritic segments [18]. The only difference between them was their morphology, which is associated with the two rat samples used: control and PILO. Further sources of distinction were introduced by two pairs of criteria that allowed us to divide dendrites into segments, distance-1/distance 2 and pruning/shrinking, resulting in 2^3^ = 8 different samples of cell models: PILO-pruned-distance 1, PILO-pruned-distance 2, PILO-shrank-distance 1, PILO-shrank distance 2, Control-pruned-distance 1, Control-pruned-distance 2, Control-shrank-distance 1 and Control-shrank-distance 2.

Our simulation results allow a prediction of what could be found in electrophysiological studies with newly born DG GCs following SE induced by PILO. The prediction is that these cells are less excitable than the control ones. Moreover, this decrease in cell excitability can be a consequence of the morphological differences between PILO and control cells alone, because in both groups we placed the same ion channel conductances, densities and distributions.

Although in our simulations the average values of the input resistances of PILO and Control cell models were not significantly different, the average value for PILO cells was slightly smaller than the one for Control cells, and this is also consistent with the prediction of smaller excitability of PILO cells.

The strategy of using different numbers of electrodes placed randomly in different dendritic regions makes it possible to evaluate the effects of the morphological differences in a more accurate way. With this strategy we evaluate how the same stimulus intensity can provoke different patterns of response depending on its distributions in the dendritic tree. Our finding that there are differences in cell response depending on both the number of electrodes and the local of stimulation confirms previous studies [29].

The major contribution of the current study is that it is possible to discriminate the level of excitability between control and PILO cells based on morphology alone. We found that depending on the dendritic region where the stimuli (electrodes) are placed, the response (firing frequency) of PILO cells is smaller than the one of control cells. This result is significant for pruned-distance 2 cell models stimulated in the IML. This result is important because it suggests that the excitability of PILO cells is highly dependent on the specific area of their dendritic trees which serve as target for synaptic input. For example, from Arisi and Garcia-Cairasco [6] it is clear that in SE induced by PILO the mossy fiber axon collaterals sprouted mostly in coincidence with the IML, where the shortest dendritic ramifications end.

It is important to mention that even though we used the same distributions of maximum ionic conductance densities and passive electrophysiological properties across dendritic segments in our cell models, because of the differences in dendritic lengths between control and PILO cells the actual dendritic surface areas reached by currents injected in the three molecular layers (IML, MML and OML) were different for the two cell groups. This implies that, effectively, the number of ionic channels activated by current injection in the same molecular layer, e.g. IML, was different from control to PILO cells (this was reflected, although not significantly, in the small difference in input resistance found between the two cell types). Moreover, this difference also depends on the segmentation criterion used, and this is why the excitability reduction was more pronounced in pruned models than in shrank models.

Therefore, even in the absence of explicit changes in the maximal ionic conductance densities, morphological alterations caused by *SE* induced by PILO may be accompanied by changes in these densities (from the point of view of the mossy fibers that innervate the IML). We propose that these changes are partly responsible for the excitability reduction in PILO cells observed in our study.

Newborn DG GCs after *SE* may also have explicit changes in the ionic channels and their density distributions [14]–[15], [27]. Because of this, there may be a coordinated effect of changes in both morphology and channel densities upon the firing behavior of DG GCs. The present study may thus be considered a first step to attempt to understand the role of morphological changes in DG GCs after SE. Therefore, future studies to investigate the possible coordinated effect between changes in both dendritic morphology and densities and distributions of ion channels on the electrophysiological behavior of DG GCs after SE are needed.

It is interesting that despite the differences observed between cells built with distance 1 and distance 2 criteria, both groups of PILO cell models showed less excitability than control groups. This finding suggests two possible scenarios in which the effects of the morphological differences can be mitigated or accentuated depending on the way in which the ionic conductances and densities are distributed. In the first, using the distance 1 criterion, for which the distribution of ionic conductances and densities did not take into consideration the dendrite length and considered only its beginning position, the excitability reduction is moderate. In the second, using the distance 2 criterion for which the distribution of biophysical properties considered the length of the dendrite there is an accentuated reduction in the cell excitability. This suggests that longer dendrites may have a more important role in the decrease of excitability than short dendrites that grow close to soma, because the differences between distance 1 and distance 2 models are concentrated in longer dendrites, which have different distance 1 and distance 2 values and, therefore, different distributions of biophysical properties.

Also, the pruning and shrinking criteria offer two possible scenarios for the effects of the morphological differences: in the first one the shorter dendrites of the PILO cells may be a product of pruning, while in the second one these dendrites may be a product of shrinking. In our simulations the pruning criterion showed more differences between control and PILO cells than the shrinking criterion. Therefore, the morphological pattern of pruned DD dendrites in PILO cell models produces decreased excitability, which could be tested in the lab using, for example, techniques of neuronal growth cone identification [30] applied in newly born DG GCs [31].

**Table 1:** Some features of dendritic morphology quantified using L-Measure [38] belonging to the IML of control and PILO cells.

	Control	PILO
**Sum of dendritic lengths**	105.8±7.6 µm	127.09±12.21 µm
**Average dendritic length**	30.15±3.8 µm	25.53±3.9 µm
**Number of branches**	2.5±0.3	4.15±0.6*
**Branch order**	1.9±0.3	2.6±0.32*
**Sum of dendritic surface areas**	122.2±20.0 µm^2^	333.87±39.98* µm^2^
**Dendritic surface area**	6.7±0.6 µm^2^	4.71±0.3* µm^2^

Values given are averages ± standard errors. The asterisk represents significant difference (*p* < 0.05).

**Table 2:** Some features of dendritic morphology quantified using L-Measure [38] belonging to the OML control and PILO cells.

	Control	PILO
**Sum of dendritic lengths**	74.76±23.05 µm	12.52±8.07* µm
**Average dendritic length**	48.82±6.14 µm	39.79±6.12* µm
**Number of branches**	2.85±0.77	0.4±0.18*
**Branch order**	2.8±0.52	1±0*
**Sum of dendritic surface areas**	139.18±44.82 µm^2^	23.97±15.76 µm^2^
**Dendritic surface area**	2.01±0.33 µm^2^	0.48±0.19* µm^2^

Values given are averages ± standard errors. The asterisk represents significant difference (*p* < 0.05).

A final commentary can be made on the potential causes of decreased excitation of the GCs in an epileptogenic circuit or network. Jakubs et al. [32] have recently shown in hippocampal slide preparations that new DG GCs born into the epileptic environment received increased inhibitory input and have a lower mean frequency of spontaneous excitatory postsynaptic currents. Also, Kobayashi and Buckmaster [14] and Otis et al. [33] have found an increase in the GABA_A_ receptors in dentate GCs in epilepsy animal models, which enhances the inhibitory effects upon these cells. In the same direction Santos et al. [10] have shown that GC after SE induced by PILO display significant reductions in spine density. All these findings match with the reduction of excitability exhibited by our computational models.

Although our computational modeling is based upon single neuron morphologies, it is clear that network modeling is needed in order to better understand the role of the newly generated cells in an environment, as close as possible to physiology. In this direction, elegant examples such as the DG network model developed by Santhakumar et al. [16], that evaluated the mossy fiber axon collaterals sprouting, or the models developed by Aimone [22] Weisz and Argibay [23], that explore the effects of neurogenesis in the DG, are excellent starting points.

Summarizing, our results predict a decrease in the excitability in PILO cells that emerge after SE. But how do we explain this kind of prediction? Why do these cells need more stimulation to reach the same response? A possible hypothesis is that this reduction in excitability due to morphological alterations is a protective mechanism that, in addition with the increase of inhibition [14], [32], [33] and reduction of spine density [10], allows the cell to be able to cope with the increased excitation that is present after SE [3]–[5], [7].

**Figure 4:**
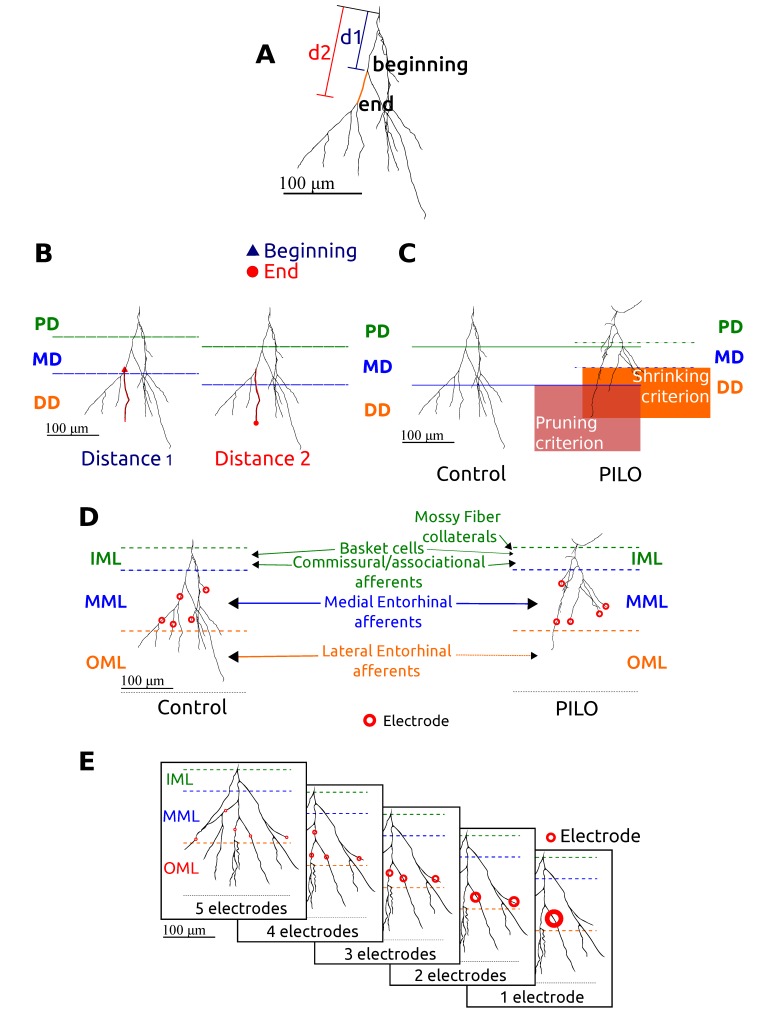
Graphical representation of the procedures used to build and stimulate the computational models. **A**. Tridimensional reconstruction of an example of cell from our sample (n20-cont02-sl1 cell from the neuromorpho database [6] showing how the two distances (d1 and d2) used to determine the distribution of biophysical mechanisms were chosen. In the figure the two distances calculated for a dendrite are represented by an orange line. **B**. Example of classification of one dendrite of the n20-cont02-sl1 cell using the two distances criteria. The dendrite represented in red was classified as a medial dendrite (MD) when using the distance 1 criterion, and as a distal dendrite (DD) when the distance 2 criterion was used. **C**. Example of utilization of the pruning-shrinking criteria to classify dendrites of the n20-cont02-sl1 and n38-r06-09-sl4 models. The horizontal lines represent the tripartite division of the average maximum values of a given distance measure for the pruned (pink area) and shrank models (orange area). Dendrites of the PILO cell that are classified as DD using the pruning and the shrinking criteria are within the pink and the orange areas respectively. Notice that with the shrinking criterion (orange area) there are more dendrites classified as DD. **D**. Schematic reconstruction of the n20-cont02-sl1 Control-GC and n38-r06-09-sl4 PILO-GC models in a general localization in the dentate granule cell layer. Laminar distributions of different afferents are shown using arrows. In the figure is shown an example of a set of five electrodes connected to five dendrites belonging to the MML, which simulate the medial entorhinal afferents. **E**. Configurations of electrode sets used to stimulate the cells. Each cell was stimulated with electrodes connected randomly to one up to five dendrites in each sublayer of the molecular layer. The figure shows an example of the stimulation protocol for current injection in sublayer MML of the Control-GC n16-cont02-sl3 model. The circle size represents the intensity of the injected current (the total intensity was the same for all 5 cases).

In this context, changes in the morphology – such as the fiber sprouting, which increases the stimulation [1], [11] in the IML – could be accompanied by changes in the dendritic tree that provoke decrease of cell excitability as a compensatory mechanism. This is in agreement with the theory of the abnormal neural networks in epilepsy [34], [35]. Therefore, it will be extremely important to evaluate the effects of this reduced excitability of newly born GC PILO cells in a network context. Thus, our next logical step would be to build a network model of the DG and to test the effects of the insertion of the new GCs with altered morphology in the circuit behavior.

**Table 3:** Passive membrane parameters, maximum ionic conductances, and reversal potentials of the channels used in the neuron models (units are given within brackets).

Parameter	Reference	Value	
*C_m_* [µF/cm^2^] (soma)	Aradi and Holmes (1999)	1	
*C_m_* [µF/cm^2^] (dendrites)	Aradi and Holmes (1999)	1.6	
*R_a_* [Ω cm]	Aradi and Holmes (1999)	210	
*Rm* [Ω cm^2^] (soma)	Aradi and Holmes (1999)	40,000	
*Rm* [Ω cm^2^] (dendrites)	Aradi and Holmes (1999)	25,000	
Leak conductance [S/cm^2^]	Aradi and Holmes (1999)	0.00004	
		**Maximum ionic conductance**	**Reversal potential [mV]**
*I*_Na_	Aradi and Holmes (1999)	0.12	45
Delayed rectifier K (Fast) [S/cm^2^]	Aradi and Holmes (2002)	0.016	−85
Delayed rectifier K (Slow) [S/cm^2^]	Aradi and Holmes (1999)	0.006	−85
A-type K [S/cm^2^]	Aradi and Holmes (2002)	0.012	−85
T-type Calcium [S/cm^2^]	Aradi and Holmes (1999)	0.000037	*
N-type Calcium [S/cm^2^]	Aradi and Holmes (1999)	0.002	*
L-type Calcium [S/cm^2^]	Aradi and Holmes (1999)	0.005	*
Ca-dependent K (SK) [S/cm^2^]	Aradi and Holmes (2002)	0.001	−85
Ca and voltage-dependent K (BK) [S/cm^2^]	Aradi and Holmes (2002)	0.0006	−85

* The reversal potential of the calcium conductances, E_ca_, was calculated at each simulation time step following the rate of change of the intracellular calcium concentration with a calcium removal rate of τ = 9 ms, and [Ca^2+^]_0_ = 70 was the resting calcium concentration [18].

## Materials and Methods

### Reconstructed Cells

We used a sample of 40 tridimensional reconstructions of newly born doublecortin-positive DG GCs, including 20 from rats that underwent SE after treatment with PILO (320 mg/kg; i.p) and 20 from control rats (treated with saline as the vehicle and with no SE) (for more details, see [6]. Whole dendritic arborizations, both apical and basal (when present), of each cell were reconstructed using Neurolucida system^©^ (MBF Bioscience). They are available at NeuroMorpho.org [36].

The two cell groups show differences in morphology, in particular in terms of the characteristics of dendrites, belonging to the three regions in which the molecular layer is divided: inner molecular layer (IML), medial molecular layer (MML) and outer molecular layer (OML) [37]. The sample cells show differences in dendritic length and surface area, and in number and order of branches of dendrites belonging to IML and OML (see Tables 1 and 2).

### Cell Models

The cell models were constructed according to Rall’s compartmental approach with membrane properties and ionic channels modeled according to the Hodgkin-Huxley formalism [28]. All simulations were run using NEURON [39], [40] with time-step of 0.025 ms and custom Matlab© (The Mathworks, Inc., Natick, MA) scripts were used for data analysis and graphs.

#### Cell models: dendritic arbor classification

Each cell in our sample had its dendritic arbor divided into segments following the canonical representation suggested by Aradi and Holmes [18], in which each GC dendrite is classified into one of four categories: dendrite of cell’s granule layer (CGL), proximal dendrite (PD), medial dendrite (MD) and distal dendrite (DD). For all cases the dendritic compartment before the first dendritic ramification from the soma was called CGL. The remainder were classified according to two distance measures between the dendrite and the soma: the first measure was the distance from soma to the beginning of the dendrite, which was called distance measure 1; and the second measure was the distance from soma to the end of the dendrite, which was called distance 2 (see Figure 4 A). We measured these two distances for each dendrite in our cell sample and calculated the average of their maximum values for the control and PILO cells. For each distance measure its average maximum value was divided into three equal parts and the three dividing points were used to classify each dendrite as a PD, a MD or a DD (see Figure 4B).

We used the average maximum values of the distance measures for the control and PILO cells to classify the dendrites into two additional patterns: in the first we used only the average maximum values of the control cells to classify control and PILO dendrites, and called this set of models the “pruned models” (see Figure 4C). The reason for this is that PILO cells have shorter dendrites and using the average maximum values of the distance measures for the control cells the number of PILO dendrites classified as DD is reduced, as if these dendrites had their ends trimmed. In the second classification pattern the average maximum values of the distance measures for PILO cells were used to classify their dendrites. We called the set of models generated by this criterion the “shrank models” based on the idea that PILO cells are shrunk versions of control cells with the same number of DD dendrites (see Figure 4C).

A total of 160 cell models were built, combining pruning-shrinking and distance 1-distance 2 criteria. Forty of them were pruned models classified using distance 1 criterion, 40 were pruned models classified using distance 2 criterion, 40 were shrank models classified using distance 1 criterion and 40 were shrank models classified using distance 2 criterion.

#### Cell models: biophysical mechanisms

Aradi and Holmes [18] described nine current types in their model of DG GC, namely: fast sodium current (I_Na_), fast and slow delayed rectifier potassium currents (I_fKDR_ and I_sKDR_), A-type potassium current (I_KA_), T, N, and L calcium current types, and BK- and SK-type calcium-activated potassium channels. Aradi and Holmes [18] also described the distribution of the maximum conductance densities of these nine current types across the four dendritic segments in which the cell’s dendritic arbor was divided.

In the current study we adopted the criterion of keeping the same nine currents (with the same kinetic parameters) used by Aradi and Holmes [18] with the same distribution of maximum conductance values across dendritic segment for both control and PILO cell models. The reason for this is that by doing so we can evaluate the effects that morphology differences alone have on the electrophysiological behavior of our cell models. Table 3 gives the values of the biophysical membrane parameters used in our cell models.

#### Characterization of the electrophysiological behavior

The 160 computational models were divided into 8 groups of 20 cells each: PILO-pruned-distance 1, PILO-pruned-distance 2, PILO-shrank-distance 1, PILO-shrank distance 2, Control-pruned-distance 1, Control-pruned-distance 2, Control-shrank-distance 1 and Control-shrank-distance 2. To characterize the cells, we worked with random samples of 10 cells taken from each one of the eight groups. Each cell model from each sample was submitted to simulated voltage clamp with 14 current values ranging from 0.005 nanoamperes (na) to 0.075 na in steps of 0.005 na. All current clamp values were injected in a consecutive way with intervals of 500 milliseconds (ms) between the end of a current injection and the start of the next.

The experimental protocol of submitting cell models to sequences of 14 current clamps was repeated three times for each cell model. In each one of them currents were applied to dendrites belonging to a different area of the DG molecular layer: IML, MML and OML (see Figure 4D). These three areas represent the laminar distribution of the different inputs that innervate GCs and the idea behind injecting current inputs in these three layers was to simulate realistic input patterns to which GCs are subject. The IML is mainly innervated by sprouted mossy fibers in epilepsy, the MML is innervated by medial entorhinal cortex (medial perforant path) and the OML by the lateral entorhinal path (lateral perforant parth). Since the published models [6] did not include the layer thickness we had to get access to the original slides and measured it for every cell.

The currents were injected using different numbers of electrodes connected randomly to one up to five dendrites in a given layer (see Figure 4E). Each current value was equally divided among the electrodes. We recorded the voltage of the cell soma along the whole stimulation and with it built plots of voltage *versus* time and frequency of spikes *versus* current (F-I curves), and also estimated the inter-spike intervals. These measurements were made for each region (IML, MML, OML) of stimulation and for each number (1–5) of electrodes.

In addition, we calculated the input resistance (R_in_) of the GC models from the slope of the steady-state current–voltage (I–V) relation from voltage responses to an injected current of −0.01 na.

The values of input resistance, number of spikes and inter-spike intervals for control and PILO groups were compared using a Student *t*-test for independent samples, with an alpha of 0.05 in all cases.
